# Evaluation of vitamin D_3_ levels and morphotic parameters of blood in prematurely born children at six years of age

**DOI:** 10.1038/s41598-019-51613-y

**Published:** 2019-10-21

**Authors:** Katarzyna Walicka-Cupryś, Katarzyna Zajkiewicz, Justyna Drzał-Grabiec, Lidia Perenc

**Affiliations:** 1 Medical Faculty, Institute of Physiotherapy, University of Rzeszow, Rejtana 16c, Rzeszów, 35-959 Poland; 2 Centre for Innovative Research in Medical and Natural Sciences, Medical Faculty, University of Rzeszow, Warzywna 1a, Rzeszów, 35-310 Poland

**Keywords:** Risk factors, Paediatric research, Nutrition, Pathology, Blood urea nitrogen

## Abstract

In Poland the preterm children, with the birth rate reaching 6.3%, constitute a serious medical problem. The system of specialistic clinics provides them with the multidisciplinary care for the first 3 years of life, including the monitoring of hematologic parameters in relation with anemia and osteopenia. The aim of this study was to assess the vitamin D_3_ level and morphotic parameters of blood in children who were prematurely born at the time when they are about to start school. The study was carried out in a group of 92 children, aged 6 years ±0.63, including 54 preterm children born at gestational age <32 weeks - and 38 full-term children. A basic anthropometric measures and assessment of morphotic parameters and the vitamin D_3_ level were assessed. Statistical analyses were carried out using Shapiro-Wilk W-test, Student’s t-test and Mann-Whitney U test. Preterm children had significantly lower the vitamin D_3_ level than controls. This also refers to some morphotic parameters of blood, such as level of hemoglobin, mean corpuscular hemoglobin concentration and platelets. Prematurely born 6–7 year-old children have significantly lower vitamin D3 levels in blood serum than normal and significantly lower hemoglobin levels than full-term children.

## Introduction

Preterm birth, or premature birth, is the birth of a baby between 22 and 37 weeks of gestational age. The preterm birth rate in Poland is 6.3%. This index is similar to other developed countries (5–10%)^1^. In Poland, preterm infants are taken care of by Neonatology Clinics or Infant Pathology Clinics. These clinics coordinate the multidisciplinary care of preterm children for the first three years of their life. The system of coordinated care includes, inter alia, monitoring metabolism of calcium and phosphorus, as well as monitoring the hematologic parameters in relation with anemia and osteopenia common in these patients^2,3^.

As the incidence of rickets is increasing both in developing and developed countries, supplementation of calcium and D3 vitamin is recommended in Poland. Vitamin D is known to regulate the intestinal absorption of calcium and phosphates. Its deficiencies lead to decrease of calcium in blood serum, while decrease of calcium in blood serum leads to parathyroid hormone secretion and consequently to the development of rickets. There is a relationship between rickets and the level of 25-hydroxyvitamin D (25(OH)D) lower than 30 nmol/l (12 ng/ml) when the decreased concentration maintains over a longer period of time. The decreased 25(OH)D denotes deficiency of D3 vitamin, yet it does not denote rickets^4^.

Preterm infants, along with infants with low birth rate, multiple pregnancy infants, and infants with decreased hemoglobin concentration, infants who lost blood in the perinatal period, and infants whose mothers had anemia during pregnancy, are given supplementary doses of iron until the 12^th^ month of age. Later, they are recommended a diet rich in iron and cow milk consumption limited to 500 ml per day^5^. There are concerns that these recommendations are not observed.

The aim of this study was to assess D3 vitamin levels and morphotic parameter of blood in children who were prematurely born at the time when they are about to start school.

## Material and Method

### Participants

This is a community-based study including 105 children aged 6 to 7 year ± 6,63, 46 boys and 59 girls. At first, we analyzed perinatal questionnaires at local hospitals and clinics and then selected children born of premature deliveries. The study population consisted of 54 prematurely born children (24 boys and 30 girls), and the control group consisted of 51 children born on time (22 boys and 29 girls). The following medical conditions and unfavorable perinatal events were found in the prematurely born children: respiratory failure (67%), respiratory distress syndrome (64%), bronchopulmonary dysplasia (29%), congenital pneumonia (11%), acquired pneumonia (24%), pneumothorax (5%), hyperbilirubinemia (82%), anemia (66%), thrombocytopenia (8%), leucopenia (8%), bleeding from respiratory or gastrointestinal system or cardiac tamponade (5%), Rhesus incompatibility in main groups (5%), periventricular leukomalacia (10%), intraventricular hemorrhage of I-II degree (50%), convulsions (11%), apnea (5%), retinopathy of prematurity (32%), TORCH infections (2%), intrauterine infections (61), sepsis (27%), purulent meningitis (22%), bacterial infection of digestive system (5%), urinary tract infection (2%), necrotizing enterocolitis (10%), gastroesophageal reflux (11%), hypoglycemia (2%), and osteopenia of prematurity (3%). The preterm infants were subject of the following medical procedures: respiratotherapy (57%), passive oxygen therapy (83%), intravenous administration of drugs (91%), parenteral feeding (82%), enteral feeding (through the probe) (53%), procedure in general anesthesia (22%), and blood or blood derivatives transfusion (64%). In some infants the following forms of congenital maldevelopment were found: patent ductus arteriosus (14%), hypoplasia of the left pulmonary artery (2%), valvular stenosis of pulmonary trunk (2%), ectopic position of the thyroid gland (2%), ectopic kidney location (2%), congenital ureteral cyst (2%), polycystic kidney disease (2%), union of the labia minora (3%), congenital deafness (2%), inguinal hernia (6%), umbilical hernia (2%), cryptorchidism (2%), hemangioma of the lower lip (2%) and lumbosacral lipoma (2%). The following criteria for including into the study group were adopted: guardians’ and children’s consent for participation, birth before gestational age of 32 weeks, lack of neurologic and orthopedic disorders on the day of the examination. Inclusion criteria for the controls were as follows: guardians’ and children’s consent for participation, lack of neurologic and orthopedic disorders, age matching that of the study group, birth at gestational age after 36 and before 42 weeks. In the control group no complications were identified during the period of adaptation after birth. At the examination day they were six years old. A preliminary power analysis was used to estimate a proper sample size with 0.95% power, α = 0.05, and expected effect size = 0.50. Based on the Central Statistical Office data, referred to children born prematurely in 2011 in the Rzeszów Municipality in Poland. The minimal required sample is 36 patients.

**Figure 1:**
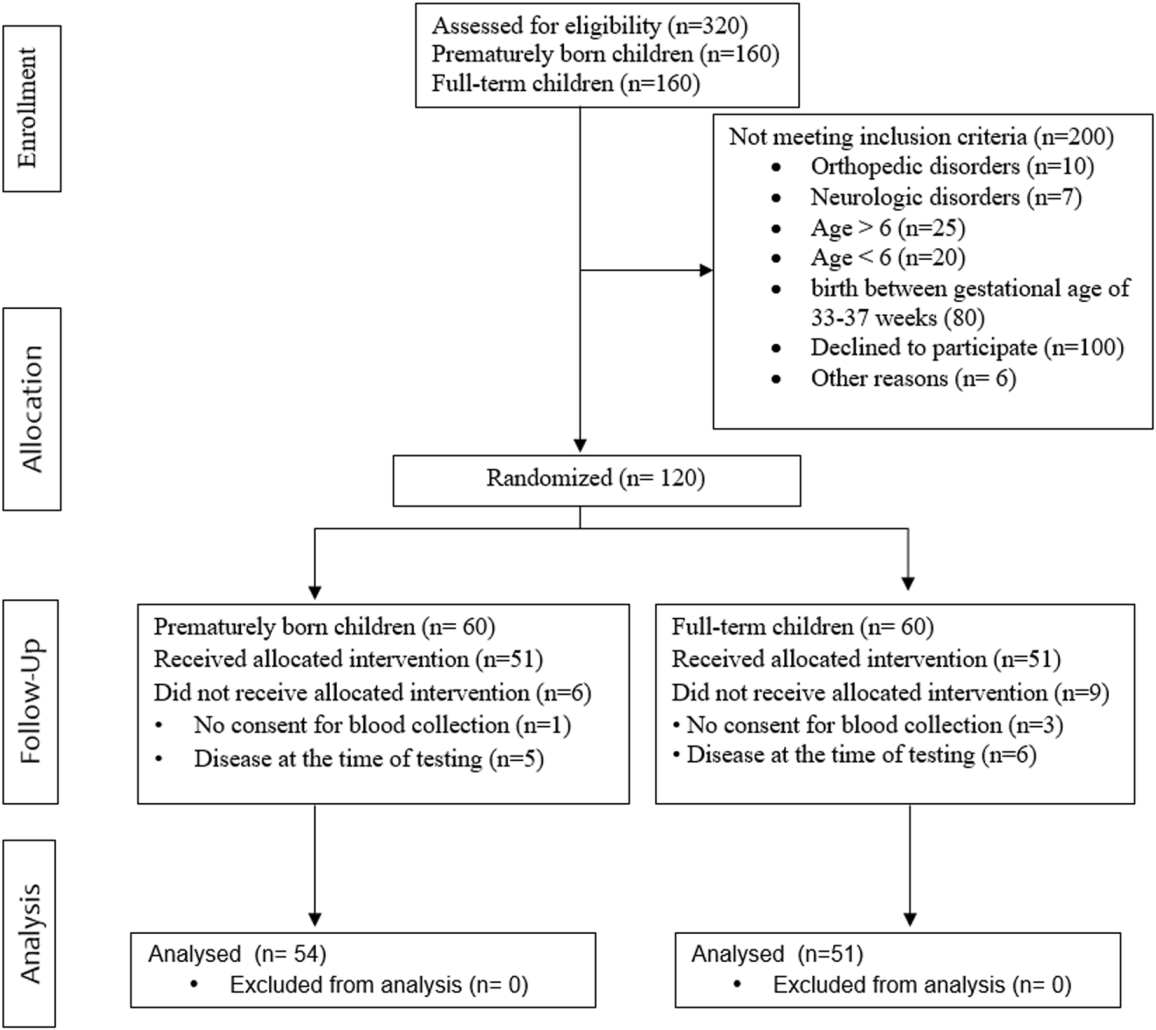
Flow diagram includes detailed information on the excluded participants – on the recruitment day.

All measurements were taken between March and May 2017 at the Institute of Physiotherapy and Centre of Medical Innovative Research being parts of University of Rzeszow. The study group consisted of children born in 2011. Out of 160 parents of preterm children and 160 parents of control group who received written invitation to participate in the study, only 105 gave their consent. The flow of children in the study is shown in Fig. 1.

The study was approved by the Medical Faculty Bioethics Commission, at the University of Rzeszów, Poland (no.6/2/2017). The children’s parents gave their written informed consent to their children’ participation in the study.

### Anthropometric measurements

All the measurements were performed on the same day, starting with anthropometric measurements. Body height was measured with Seca 213 mobile stadiometer, with an accuracy of 0.1 cm. Body mass was measured using electronic scale OMRON BF 500, with an accuracy of 0.1 kg. The measurements were performed in standard conditions; children in underwear and barefoot, were standing in upright position, without bending knees. In case of all children, basic anthropometric measures were performed and Body Mass Index (BMI) was calculated. Anthropometric measures of both groups are shown in Table 1.

**Table 1:** Anthropometric parameters in the studied groups.

Variables	Preterm children (n = 54)			Control group (n = 51)			Z	*p*
	$\bar{x}$	Me	SD	$\bar{x}$	Me	SD		
Body height	116.52	115.50	8.02	118.98	118.00	7.91	−1.82	0.069
Body mass	21.11	19.90	4.78	23.12	21.90	4.74	**−2.57**	**0.010**
BMI	15.40	15.11	2.05	16.20	15.80	1.94	**−2.35**	**0.019**

$\bar{x}$-arithmetic mean; Me-median; SD-standard deviation; Z-result of Mann-Whitney U test; p-level of probability.

Only in 92 cases, the parents gave permission for taking the blood samples from their children. Assessment of morphotic parameters was performed with the use of the CellDyn 1700 device (Abbott Laboratories, USA) and vitamin D_3_ levels in serum was assessed with the use of the VIDAS® 25-OH Vitamin D Total (bioMerieux, France). The VIDAS® 25-OH Vitamin D Total assay is therefore considered suitable for assessment of vitamin D status in clinical routine.

All methods were performed in accordance with the relevant guidelines and regulations.

### Data analysis

We conducted statistical analysis of the collected data with the use of the Statistica 13.5 (Stat Soft, Poland). To analyze the variables, we used both parametric and nonparametric tests. The choice of a parametric test was conditioned by meeting its basic assumptions, i.e. whether the distribution of the studied variables was in accordance with the normal distribution. This was verified with the Shapiro-Wilk test. For all numerical data, we calculated its descriptive statistics: the mean, the median and the standard deviation. We used the Student t-test for independent variables to assess the differences in the mean value of the numerical features in the two groups studied, or, alternatively, we used the nonparametric Mann-Whitney U test. Statistical significance was assumed at p < 0.05.

## Results

We found statistically significant differences for the following parameters: Hemoglobin (Hgb) (p = 0.015), Mean Corpuscular Hemoglobin Concentration (MCHC) (p < 0.001) and D3 (p < 0.001). All these parameters were higher in the clinical control group (Table 2).

**Table 2:** Morphotic parameters and the D vitamin level.

Variables	Preterm children (n = 54)			Control group (n = 38)			t/Z	*p*
	$\bar{x}$	Me	SD	$\bar{x}$	Me	SD		
WBC	8.82	8.55	2.50	8.45	7.60	2.30	0.57	0.568
RBC	4.73	4.72	0.31	4.85	4.86	0.30	*−1.75*	*0.082*
HGB	12.89	13.10	0.71	13.30	13.30	0.76	**−2.43**	**0.015**
MCV	82.69	82.00	3.86	81.68	82.10	3.22	0.79	0.432
MCH	27.29	27.00	1.60	27.47	27.55	1.10	−1.24	0.216
MCHC	33.00	32.90	0.97	33.65	33.70	0.37	**−4.76**	**<0.001**
PLT	352.44	342.00	93.66	369.39	343.00	90.01	−1.01	0.314
MPV	9.28	9.05	1.55	8.88	8.80	0.87	0.92	0.359
D3	16.92	14.60	7.90	22.55	22.00	4.88	**−3.81**	**<0.001**

n- number of observations; $\bar{x}$- arithmetic mean; Me-median; SD- standard deviation; t- result of Student’s t-test for independent variables; Z- result of Mann-Whitney U test p- level of probability; WBC - White Blood Cell, RBC - Red Blood Cell; HGB - Hemoglobin, MVC - mean corpuscular volume; MCH - Mean Corpuscular Hemoglobin; MCHC - Mean Corpuscular Hemoglobin Concentration; MCV - Mean Corpuscular Volume; MPV - Mean Platelet Volume; PLT – Platelets; D3 – 25-hydroxyvitamin D.

There were statistically significant differences in the two groups of girls for the following parameters: Mean Corpuscular Volume (MCV) (p = 0.017), MCHC (p = 0.001), Platelets (PLT) (p = 0.028) and D3 (p = 0.005). The MCV parameter was higher in preterm girls, while the MCHC, PLT and D3 parameters were higher in the control group (Table 3).

**Table 3:** Morphotic parameters and the D vitamin level according to gender.

Variables	Preterm girls (n = 30)			Control girls (n = 19)			t/U	*p*
	$\bar{x}$	Me	SD	$\bar{x}$	Me	SD		
WBC	9.13	8.65	2.62	8.77	7.60	2.58	264.0	0.677
RBC	4.74	4.71	0.30	4.89	4.82	0.33	*−1.63*	*0.109*
HGB	12.94	13.10	0.74	13.20	13.20	0.79	225.0	0.224
MCV	82.98	82.35	3.56	80.48	80.40	3.22	2.48	**0.017**
MCH	27.35	27.20	1.37	27.05	26.80	1.03	*0.83*	*0.408*
MCHC	32.98	32.90	1.03	33.61	33.60	0.36	124.0	**0.001**
PLT	333.73	319.00	79.12	390.37	344.00	97.18	178.0	**0.028**
MPV	9.24	9.10	1.08	8.90	8.80	0.77	*1.18*	*0.244*
D3	16.80	13.50	8.42	22.42	22.00	5.91	152.5	**0.005**
**Variables**	**Preterm boys (n = 24)**			**Control boys (n = 19)**			**t/U**	**p**
	$\bar{x}$	**Me**	**SD**	$\bar{x}$	**Me**	**SD**		
WBC	8.43	7.55	2.34	8.12	8.10	1.98	*0.46*	*0.645*
RBC	4.73	4.72	0.33	4.81	4.87	0.27	*−0.85*	*0.401*
HGB	12.82	12.90	0.67	13.40	13.30	0.72	−2.72	**0.009**
MCV	82.33	81.55	4.26	82.87	83.00	2.81	172.0	0.177
MCH	27.22	26.75	1.87	27.90	27.70	1.03	127.0	**0.013**
MCHC	33.03	32.90	0.91	33.68	33.70	0.39	88.5	**<0.001**
PLT	375.83	359.00	106.28	348.42	341.00	79.26	190.5	0.363
MPV	9.34	8.75	2.02	8.87	8.60	0.98	211.0	0.690
D3	17.07	17.10	7.38	22.68	22.00	3.74	−3.02	**0.004**

$\bar{x}$- arithmetic mean; Me-median; SD- standard deviation; *t- result of Student’s t-test for independent variables*; Z- result of Mann-Whitney U test p- level of probability; WBC - White Blood Cell, RBC - Red Blood Cell; HGB - Hemoglobin, MVC - mean corpuscular volume; MCH - Mean Corpuscular Hemoglobin; MCHC - Mean Corpuscular Hemoglobin Concentration; MCV - Mean Corpuscular Volume; MPV - Mean Platelet Volume; PLT – Platelets; D3 – 25-hydroxyvitamin D.

For boys, we found statistically significant differences in the following parameters: Hgb (p = 0.009), Mean Corpuscular Hemoglobin (MCH) (p = 0.013), MCHC (p < 0.001) and D3 (p = 0,004). All these parameters were higher in the control group of boys (Table 3).

## Discussion

Preterm birth is associated with a prolonged period of adaptation and with numerous adverse perinatal events. This may cause nutritional and metabolic complications that persist long−term. This is confirmed by the results of the present study. We found that the preterm children had significantly lower vitamin D3 levels than the controls. Vitamin D3 level in the preterm children also was significantly lower than universal norms. In assessment of morphotic parameters of blood we found significant differences in hemoglobin level parameters - these parameters were significantly lower in preterm children in their sixth year of age.

In view of the above, the authors of this study believe that in the case of prematurely born children it is necessary to perform routine examinations assessing morphotic parameters of blood and vitamin D_3_ levels at least once in 12 months, until the age of 7 years. Furthermore, if any abnormalities are identified, such as anemia or vitamin D_3_ deficiency, more comprehensive diagnostic examinations should be conducted in order to administer an adequate therapy.

As shown by another study^6^ involving a similar group of subjects, i.e. preterm children about to start school, the combined number of unfavorable perinatal events differentiates umbilical skinfold thickness and global adiposity. Higher combined numbers of unfavorable perinatal events correspond to lower values of umbilical skinfold thickness and global adiposity. It was also demonstrated that lower gestational age at birth corresponded to higher combined numbers of unfavorable perinatal events. At the same time, regarding some health-related aspects, there are no differences between pre-term and full-term children^7^.

Our findings can be confirmed by the literature. Still, studies conducted so far have focused on the discussed parameters in babies. Until now, no studies have been conducted on older children. Very preterm infants had risks of developing vitamin D deficiency compared with full-term infants but the association between gestational age and vitamin D status remained unclear^8,9^. According to Park *et al*., 98.9% of preterm infants had vitamin D insufficiency or deficiency, and 51.1% of preterm infants were severely vitamin D deficient^8,10^. Burris *et al*. found that, compared with more mature infants, those born before 32 weeks’ gestation age had higher odds of umbilical cord plasma 25(OH)D levels below 20 ng/ml^8^. Currently, there is limited information on the distribution of 25(OH)D levels in preterm infants. A few studies have documented 25(OH)D levels from infants at birth with sample sizes ranging from 8 to 34^11–15^ with mean 25(OH)D levels ranging from 16.3 nmol/ml (~6.5 ng/ml) among preterm infants born to women in the United Arab Emirates^15^ to 29.2 nmol/L (~10 ng/ml) in Finland^14^. Mimouni *et al*. suggest that each extremely preterm infant should be monitored for adequate vitamin D status^16^.

Considering that maternal vitamin D status is the most important factor determining vitamin D status at birth it was predicted that the incidence of vitamin D deficiency would be higher in early preterm infants^17–19^.

Some studies reported low blood hemoglobin levels in preterm infants. Matysiak believes that preterm infants had risks of anemia as they had iron deficiencies. Therefore, iron supplementation is advised^20^. From the second week of a baby’s life its hemoglobin concentration decreases to reach its low between the second and the third month. This is known as physiological anemia of the first quarter. It is a physiological phenomenon and does not require treatment. It is caused by erythropoietin insufficiency, shortened erythrocyte life cycle and increased hemolysis. Lower red cell parameters are found in a healthy premature infant as long as till the second or third month, as compared with healthy full-term infants. In preterm infants, physiological anemia develops earlier, hemoglobin concentration starts decreasing in the fifth day of life and lasts for five to eight weeks reaching its low in the seventh week of infants’ life. The greatest hemoglobin decreases are found in the smallest and most premature infants^21^.

According to some authors, anemia is commonly seen in preterm infants^22–24^. Until now, there have been no studies on whether infant anemia results in low hemoglobin levels in older children. There are also reports suggesting that very preterm infants may have fewer platelets as a result of immaturity of their bone marrow and liver. They also may have fewer of the large and the most active platelets. In comparison to full-term infants, preterm infants have less P-selectin^25,26^, their platelets are more hyporeactive, and less able to aggregate^27^. However, our study has not confirmed such differences, possibly due to a small fraction of very preterm children in our study population.

Nutrition is one of the sources of vitamin D for humans. It is believed, however, that only approximately 10% of vitamin D used by the body is exogenous in origin^28^. According to the latest Polish guidelines related to prophylactic dosing of vitamin D and therapy due to its deficiency, in the case of healthy children aged 1–10 years who are exposed to sunlight, with uncovered forearms and legs and without sunscreen for a minimum of 15 minutes, between 10 a.m. and 3 p.m., from May to September, supplementation is not necessary although it is recommended and safe. If the above conditions are not met, supplementation of 600–1000 IU/day throughout the year is recommended, depending on body weight and dietary vitamin D intake^29^. Research has shown that the diet of Polish children aged 4–6 years is similar to that observed in the same age group in various parts of Europe, and more than 98% of children do not take in the recommended dose of vitamin D_3_ with their diet^30^. This low vitamin D intake is typical for pre-school age children both in Poland^31,32^ and in other countries^33,34^. Obviously, in addition to dietary sources, vitamin D is also synthesized by skin during exposition to ultraviolet radiation^35^. At the geographical latitude of Poland, between 54° and 49°N, the total annual insolation is low to medium, amounting to 1600 h^36^. In the summer (June, July) the optimum vitamin D synthesis occurs for nine hours, while in spring (March) and autumn (September) it is only for three hours daily, and during winter months the figure is still lower. Due to this, the significant deficiency of dietary vitamin D intake, as observed in the children, cannot be compensated for by the synthesis in the skin.

The value of our study is the study population of six-year-old children who were born before 32 weeks of gestational age. So far, all analyses were conducted on populations of babies. Additionally, we measured the basic morphotic parameters in the same study population - this has not been studied before either. Our study is the first complex study on a population of preterm born children who are about to start school.

The limitation of our study is the small size of the study population as well as lack of regression analysis with respect to the gestational age.

## Conclusion

Our data showed that prematurely born six-year-old children have significantly lower vitamin D3 levels in blood serum, and these levels are below the normal range. Also, the prematurely born children have significantly lower hemoglobin levels than full-term children.

## Data Availability

The authors of the manuscript shall provide “Data Availability,” if it is necessary (see: New Zealand Clinical Trials Registry (ANZCTR): ACTRN12618001948280).
